
JOURNAL INFORMATION
==============================
NLM Title Abbreviation: Tex Med J (Austin)
NLM Journal ID: 21130590R

Texas Medical Journal

ISSN: 0892-8495
Publisher: Biomedical Journal Digitization Project

ARTICLE INFORMATION
==============================
PMCID: PMC9626132
PMID: 36956328
Article version: 1
Subjects: Correspondence

Dr. Shufeldt’s “The Negro—A Menace to American Civilization”

Keane A. H. LL. D. Late Vice-President Royal Anthropoligical Institute. Arám Gáh, 79 Broadhurst Gardens, South Hamstead, London, N. W.

Print publication date: 1908 Jun
Volume: 23
Issue: 12
First page: 511
Last page: 516
Page range: 511-514, 516
License: This work is free of known copyright restrictions. For more information, please see PMC Back Issue Digitization.
License URL: https://creativecommons.org/publicdomain/mark/1.0/

==============================

### Dr. Shufeldt's "The Negro—A Menace to American Civilization."

LONDON, ENGLAND, May 7, 1908.

*To the Editor Texas Medical Journal.*

SIR: May I, as an outsider, but not an "alien," crave a little of your valuable space for a few remarks on this work, to which my attention has recently been called as again raising the whole question of "the black eyesore" in an acute form. Equipped as he is with all the armature acquired from his ethnological and anatomical studies, and from long and close contact with the negroes, the author speaks at first hand with a commanding voice that few others could pretend to. Hence, when he tells us that in writing the book he has been actuated by no other motive than that of "telling the truth most plainly and fully, his conclusions on the many complex factors of the problem will at least be taken into serious consideration, though they may not succeed in securing universal assent. The issues raised are too numerous and involved to hope for an immediate solution, and for he present all that can be reasonably expected is some such full and frank statement of the case as is here presented to the reader.

In support of Dr. Shufeldt's general argument, I should like here to dwell more particularly on two points—*the negro's intellectual stagnation* and the effects of miscegenation—on both of which I may claim to speak with some confidence, if not altogether convincingly.

I. I believe I was among the first anthropologists to enter a strong protest against the assumptions of those philanthropists and sham sentimentalists who on political, social and religious grounds rashly proclaimed the fundamental equality of all the races, and preached the doctrine that the Zulu-Kaffirs and other Africans stood on the same mental level as the Aryan-speaking white peoples. So far back as the year 1884 I pointed out in the Encyclopedia Britannica (9th edition) that such equality was belied by the history of human culture, and disproved by the physical structure of the black man's brain cap. "The cranial sutures close much earlier in the negro than in other races." To this premature ossification of the skull, preventing all further development of the brain, many pathologists have attributed the inherent mental inferiority of the blacks, an inferiority which is even more marked than their physical differences. No one has more carefully studied this point than Filippo Manetta, who, during a long residence on the plantations of the Southern States of America, noted that the negro children were sharp, intelligent and full of vivacity, but in the adult state the intellect seems to become clouded, animation giving place to a sort of lethargy, briskness yielding to indolence. With the white the volume of the brain grows with the expansion of the skull; with the negro the growth of the brain is, on the contrary, arrested by the early closing of the cranial sutures. (*La Razza negra,* Turin, 1864, p. 20.) Later the same phenomena, causing stagnation of both the mental and moral faculties, was

UNEQUALED FOR DELICACY OF FLAVOR AND NUTRITIOUS PROPERTIES

**PHILLIPS' DIGESTIBLE COCOA**

"THE ONLY COCOA WITH A RICH CHOCOLATE FLAVOR"

**A VALUABLE SUBSTITUTE FOR TEA AND COFFEE**

Differing from other Cocoas, directions for preparation must be carefully followed when an easily digested food beverage will result.

WHERE LIQUID NOURISHMENT IS IMPERATIVE

IT WILL BE FOUND

NOURISHING AND SUSTAINING

**PHILLIPS' MILK OF MAGNESIA**
**PHILLIPS' COD LIVER OIL EMULSION**
**PHILLIPS' PHOSPHO-MURIATE OF QUININE**

noted by other observers such as Colonel A. B. Ellis and Captain Binger, the latter declaring that "this intellectual arrestment agrees with what we see in these regions (Soudan); through the sealing up of the brain cavity the development of the skull is arrested, and the brain does not develop to advantage." (Quoted in my *Ethnology,* page 44.) Moral, no less than the purely intellectual qualities remain at the animal stage is evident from the unbiased testimony of such observers as Captain Ruffin, of Richmond, Va., and especially the Rev. Dr. Tucker, who stated at the American Church Congress for 1883 that he "knew of whole neighborhoods where there is not one single negro couple, whether legally married or not, who are faithful to each other beyond a few weeks. * * * The most pious negro that I know is confined for an atrocious murder, and he persists in saying that he can see no offense against God in his crime, though he acknowledges an offense against man." He had not yet grasped the distinction between *sin* and *crime.* His moral faculties were still infantile, and it is this very infantile condition of the negro temperament, combined with an ineradicable strain of animal ferocity, that makes the negro element such a "menace to American civilization."

I may incidentally remark that the cranial capacity on which the mentality depends stands in Soudan at about 1300 cc., as compared with Dubois' *Pithecanthropus erectus* (900 or perhaps 1000), and the average Anglo-American (1600). The average negro is thus intellectually about midway between the human precursor (early Pliocene men) and the present most advanced civilized people. In other words, he lags in terms of time at least one million years behind. Such is the inference to be drawn from the data supplied by physical anthropology.

II. Racial temperaments resemble physical temperatures in that the union of the two of either results in equilibrium. Hence the union, the intermingling, of two races of different cultural level means the relative upheaval of the lower at the expense of the higher. Consequently, the "Afro-American," now much spoken of, is necessarily a debased creature compared with the typical Anglo-American of more or less pure "Anglo-Saxon" or British descent. It follows that if this Afro-American element is on the increase, the Anglo-American element has entered on a process of decay which must end in all-round debasement unless arrested. Can such a dismal prospect be looked upon with equanimity by that "Little American" section of the community which, like our (I fear) still more numerous "Little Englanders" have always the first word for the alien, the second only for themselves? For it is to be noticed that the degradation would not be confined to the Southern States, but would inevitably spread like a pestiferous contagion over the whole union. Indeed, it already threatens to spread, and a silent northward movement appears to have recently set in, the Southern blacks even now pouring into the city of New York, as I am informed, not by alarmists, but by cool-headed observers here. Of this aspect of the question I read almost with terror the warning note raised by Dr. Shufeldt, who tells us that "there are plenty of people in this country of ours who would far rather see the entire white race here robbed by heroic injections into their veins of all the saveragery and criminality there is in the negro than have any number of the latter in any way inconvenienced by their being returned to the country from which their ancestors came." Such fanatical regard for the susceptibilities of a race, which, after all, is entitled to scant respect, becomes a crime against humanity, and, if persisted in, would end in national suicide. Surely they can not shut their eyes to the deadly result of miscegenation in Latin America, although here the crosses have mainly been between the whites and Americans, the latter being in most respects a far superior race to the blacks, so that the resultant Hispano and Lusitano-American Meztizos must be assumed to be of better type than the Afro-Americans. Dealing with the various degrees of intermixture, Sir Harry Johnson aptly remarks that the white and black stock "are too widely separated in type to produce a satisfactory hybrid." This observer, who has had more to do with the African aborigines than any other man living, also holds that the pure and unadulterated negro can not advance with any certainty of stability above his present level of culture without admixture of a somewhat higher race, such as the Indian coolie. Then the blend of these two races "would give the Indian the physical development which he lacks, and he in his turn would transmit to his half-negro offspring the industry, ambition and aspiration toward the civilized life which the negro so markedly lacks." (Quoted in my *Man Past and Present,* page 265.) The infusion of such a Hindu strain has even been suggested as a possible means of improving or elevating the North American negro. But I do not consider such a product as at all practical and hold with Dr. Shufeldt that the only ultimate remedy for the growing evil must be "separation," or in plain removal or excision from the body politic. But a consideration of the various schemes by which it is proposed to effect this and thereby disperse the "black cloud" does not fall within the scope of the present communication. I am, sir,

Yours faithfully,

[Signed] A. H. Keane, LL. D.,
Late Vice-President Royal Anthropoligical Institute.
Arám Gáh, 79 Broadhurst Gardens,
South Hamstead, London. N. W.

---

Dr. U. E. G. Dyer has removed from Houston to Ballinger, and limits his practice to diseases of the eye, ear, nose and throat. He has just returned from Chicago and New York, where he has completed a course of special instruction.

---

### Abstracts and Selections.

### A Few "Buts."

"Just keep your heart a beating warm,
Be kind to every fellow;
Look for rainbows in the storm,—
But carry an umbrella."
